# circ-ZEB1 Enhances NSCLC Metastasis and Proliferation by Modulating the miR-491-5p/EIF5A Axis

**DOI:** 10.1155/ancp/5595692

**Published:** 2025-01-04

**Authors:** Qi Wang, Shengying Ling, Jia Lv, Lina Wu

**Affiliations:** ^1^Department of General Practice, Renji Hospital, Shanghai JiaoTong University School of Medicine, Shanghai, China; ^2^Department of Obstetrics and Gynecology, Shanghai Fourth People's Hospital, School of Medicine, Tongji University, Shanghai 200434, China

**Keywords:** circ-ZEB1, EIF5A, miR-491-5p, non-small cell lung cancer (NSCLC)

## Abstract

**Background:** Circular RNAs (circRNAs), covalently closed single-stranded RNAs, have been implicated in cancer progression. A previous investigation revealed that circ-ZEB1 is expressed abnormally in liver cancer. However, the roles of circ-ZEB1 in non-small cell lung cancer (NSCLC) are unknown.

**Methods:** In this study, we used fluorescence in situ hybridization (FISH) and RT-qPCR to study circ-ZEB1 expression in NSCLC cells and tissues. A luciferase reporter assay was performed to validate downstream targets of circ-ZEB1. Transwell migration, 5-ethynyl-20-deoxyuridine (EdU), and cell counting kit-8 (CCK8) assays were performed to assess proliferation and migration. In vivo metastasis and tumorigenesis assays were also performed to investigate circ-ZEB1 functions during NSCLC.

**Results:** Our results showed that circ-ZEB1 expression was increased in NSCLC tissues and cells. circ-ZEB1 downregulation suppressed NSCLC cell proliferation as well as migration in vitro and in vivo. Luciferase data confirmed EIF5A and miR-491-5p as downstream targets of circ-ZEB1. EIF5A overexpression and miR-491-5p suppression reversed NSCLC cell migration post circ-ZEB1 silencing.

**Conclusion:** Our collective findings advised that circ-ZEB1 takes part in the malignant progression through regulating the miR-491-5p/EIF5A axis, highlighting its potential as an effective NSCLC therapeutic target.

## 1. Introduction

Lung cancer is a general malignancy characterized by high incidence and poor prognosis; non-small cell lung cancer (NSCLC) is a general class of lung cancer, comprising approximation 85% [1]. Lung cancers can be divided into two groups, small cell lung carcinoma (SCLC) and NSCLC, based upon morphology differences in tumor cells [2]. A minority of NSCLC patients are diagnosed at an early stage and can be treated with surgery, while the majority (>60%) of NSCLC patients are only eligible for traditional radiation and chemotherapy and have a poor prognosis [2]. Thus, the use of biomarkers to investigate the abnormal human internal environment has attracted wide attention.

Circular RNAs (circRNAs) are endogenous single-stranded RNAs that form a covalently closed loop and do not have 3′ and 5′ polar ends, which makes them less susceptible to degradation than linear RNA [3–5]. Numerous studies have confirmed that circRNAs play an important role in the progression of various tumors, including breast cancer, gastric cancer, gastric cancer, and hepatocellular carcinoma [6–9]. circNDUFB2 has been shown to suppress NSCLC by destabilizing IGF2BPs and activating antitumor immunity [10]. The oncogenic circRNA C190 promotes NSCLC through EGFR/ERK pathway modulations [11]. circRNA_100876 downregulation suppressed NSCLC progression in vitro by targeting miR-636 [3]. circ-ZEB1 has also been shown to enhance PIK3CA expression through miR-199a-3p silencing, which affects hepatocellular carcinoma proliferation and apoptosis [12]. However, the function of circ-ZEB1 in NSCLC progression is unclear. So, the aim of this study was to unravel the role of circ-ZEB1 in NSCLC progression.

In the present study, we found that circ-ZEB1 is upregulated in both NSCLC tissues and cells and that it has a negative positive with the migration and invasion of NSCLC cells. Mechanistically, circ-ZEB1 can regulation the malignant progression of NSCLC by miR-491-5p/EIF5A signaling pathway. Revealing circ-ZEB1 as a potential novel effective target for NSCLC treatment.

## 2. Materials and Methods

### 2.1. Ethics Statement

Four-week-old BALB/c nude female mice (15–20 g) were obtained from SLARC (Shanghai, China). The Animal Research Committee at Shanghai Renji Hospital, Shanghai Jiao Tong University approved animal protocols.

### 2.2. Tissue Chips

Both cell and NSCLC tissue (*n* = 30 from Shanghai Renji Hospital, Shanghai Jiao Tong University) were used with probes for circ-ZEB1 expression. For fluorescence in situ hybridization (FISH), nuclei were counterstained with DAPI (Yeasen Biotechnology, Shanghai, China) for 15 min. Images were obtained with a Zeiss LSM 700 confocal microscope (Carl Zeiss GmbH; Oberkochen, Germany).

### 2.3. Cell Culture

The NSCLC cell lines NCI-H1299, H1650, PC9, and A549, as well as a normal lung cells Beas-2B, were acquired from the Shanghai Cell Bank in Chinese Academy of Sciences. Cells were cultured in DMEM (Gibco; Thermo Fisher Scientific) with 10% fetal bovine serum (FBS; Gibco; Thermo Fisher Scientific).

### 2.4. RNA Overexpression or Interference

EIF5A overexpression vector through putting EIF5A cDNA to pcDNA3.1 vector. MiR-491-5p suppressor, miR-491-5p mimics, and circ-ZEB1 siRNA were synthesized via Genepharma (Suzhou, China). Transient transfections were performed using Lipofectamine 2000 transfection reagent (Invitrogen, Carlsbad, CA, USA).

### 2.5. Bioinformatics Analysis

Correlations between miRNA, mRNA, and circRNA were predicted via http://starbase.sysu.edu.cn/.

### 2.6. Cell Migration Assay

Twenty-four-well transwell chambers (BD Biosciences, NJ, USA) were used for cell migration analyses. PC9 and A549 cells were plated into the upper chamber and 500 µL of DMEM with 20% FBS was added to the lower chamber. We cultured cells for 1 day at 37°C before being fixed to the bottom chamber cells for 0.5 h with 4% paraformaldehyde. Cells were then stained with 0.1% crystal violet (Shanghai Yisheng Biotechnology, Shanghai, China) and images were obtained with a Zeiss Axio Observer D1 microscope.

### 2.7. 5-Ethynyl-20-Deoxyuridine (EdU) Analysis

EdU assay kits (Thermo Fisher Scientific) were used to analyze cell activity. PC9 and A549 cells (1 × 10^5^) were seeded in six-well plates for 2 days prior to the addition of EdU for 2 h. Cells were fixed with 4% formaldehyde.

### 2.8. Cell Counting Kit-8 (CCK8) Assay

PC9 and A549 cells were incubated in 10% CCK8 diluted in normal culture medium at 37°C. Proliferation rates were recorded after 0, 1, 2, and 3 days of transfection. The absorbance of each was recorded with a microplate reader.

### 2.9. RT-qPCR

RNA from tumor tissues and cells was isolated and cDNA was synthesized with the TaqMan miRNA reverse transcription kit (Thermo Fisher Scientific). The 2^−*ΔΔ*Ct^ method was used to capture fold changes in relative expression. The following primers were used: circ-ZEB1: 5′-TGTGGGGTGTGAGAACTTGA-3′ (forward) and 5′-ATGCTGCTTTGACAGGGTTT-3′ (reverse); miR-491-5p: 5′-GGAGTGGGGAACCCTTCC-3′ (forward) and 5′-GTGCAGGGTCCGAGGT-3′ (reverse); EIF5A: 5′-ATTTGAAACGCTGGAGACT-3′ (forward) and 5′-CCCATCATGCCTGTCAGTA-3′ (reverse); U6: 5′-CTCGCTTCGGCAGCACA-3′ (forward) and 5′-AACGCTTCACGAATTTGCGT-3′ (reverse); GAPDH: 5′-TGTGGGCATCAATGGATTTGG-3′ (forward) and 5′-ACACCATGTATTCCGGGTCAAT-3′ (reverse).

### 2.10. Dual Luciferase Reporter Assay

Putative miR-491-5p-binding sites of the 3′ untranslated region (UTR) of *EIF5A* and wild type (Wt) or mutant (Mut) circ-ZEB1 were cloned into the psi-CHECK vector (Promega, WI, USA); the clones were referred to as EIF5A-Wt/circ-ZEB1-Wt and EIF5A-Mut/circ-ZEB1-Mut. Firefly luciferase or circ-ZEB1 3′ UTR was the primary luciferase signal and Renilla luciferase was used for normalization. A549 cells were transfected with Lipofectamine 2000 and firefly and Renilla luciferase activity was detected 1 day after transfection.

### 2.11. Tumor Xenograft Formation

We injected viable Wt or 2 × 10^6^ sh-circ-ZEB1 A549 cells into the right flank of mice and tumor sizes were measured every 5 days for 1 month using a Vernier caliper. Tumor volume was determined using the following formula: length × width^2^ × 0.5. The relative expression of Ki67 was measured with the IH method.

We stably transfected luminescence-labeled A549 (Luc-A549) cells with negative control (NC) for metastasis analyses. Wt or 2 × 10^5^ sh-circ-ZEB1 A549 cells injected into nude mouse tail veins. After 4 weeks, lung metastasis was validated using a bioluminescence imaging system. Lung tissue metastatic foci were counted after hematoxylin and eosin (HE) staining.

### 2.12. Statistical Analysis

Statistics analysis was conducted with GraphPad Prism Software (GraphPad, San Diego, CA, USA). Statistician determined statistical differences using Student's *t*-test for comparisons between two groups and by ANOVA for comparisons among three or more groups. Data are denoted by means ± standard deviation (SD). *p*-values ≤ 0.05 are regarded statistical significance.

## 3. Results

### 3.1. circ-ZEB1 Expression was Increased in NSCLC Tissues and Cells

**Figure 1:**
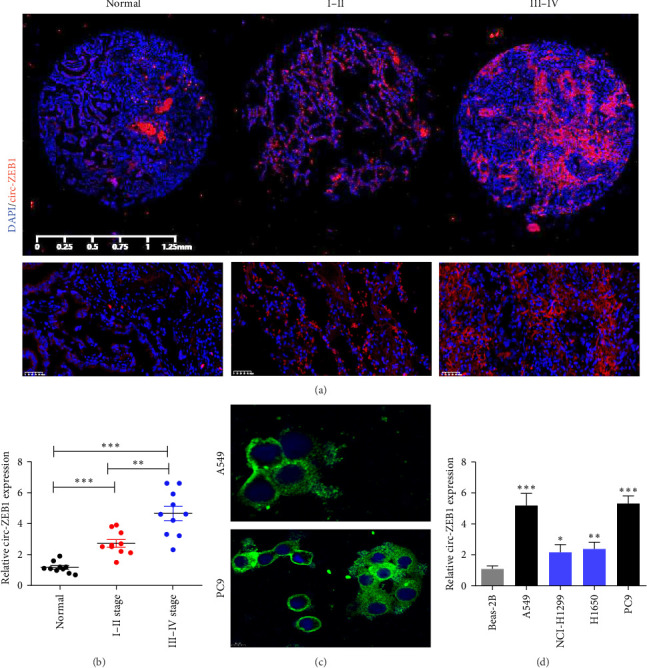
Expression of circ-ZEB1 was increased in both non-small cell lung cancer (NSCLC) tissues and cell lines. (A and B) FISH analysis shows the expression and subcellular localization of circ-ZEB1 in 30 pairs of NSCLC cancer and adjacent tissues. Data are expressed as mean ± standard deviation (SD). *⁣*^*∗∗*^*p* < 0.01 and *⁣*^*∗∗∗*^*p* < 0.001 versus normal. (C) FISH analysis shows the expression and subcellular localization of circ-ZEB1 in NSCLC cells. (D) RT-qPCR shows the expression of circ-ZEB1 in normal Beas-2B cells and the NSCLC cell lines A549, NCI-H1299, H1650, and PC9. Data are expressed as mean ± SD. *⁣*^*∗*^*p* < 0.05, *⁣*^*∗∗*^*p* < 0.01, and *⁣*^*∗∗∗*^*p* < 0.001 versus Beas-2B.

FISH analysis revealed that circ-ZEB1 expression was increased in NSCLC tissue (Figure 1A). Statistical analysis confirmed that circ-ZEB1 expression was increased in III–IV stage cancer tissues (Figure 1B), suggesting that higher expression of circ-ZEB1 may be linked to tumor invasion. FISH analysis also showed that circ-ZEB1 was localized in the cytoplasm of PC9 and A549 cells (Figure 1C). RT-qPCR showed that circ-ZEB1 expression was increased in NSCLC cells NCI-H1299, A549, H1650, and PC9 compared with Beas-2B cells. Both A549 and PC9 cells showed higher circ-ZEB1 expression; these cell lines were selected for subsequent experiments (Figure 1D).

### 3.2. Downregulation of circ-ZEB1 Inhibits NSCLC Cell Proliferation and Tumor Growth

**Figure 2:**
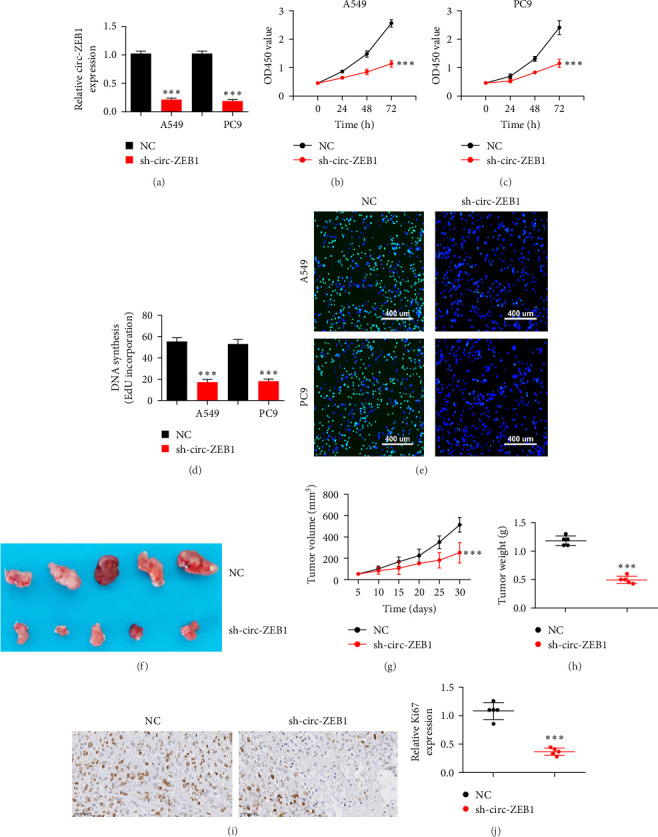
Downregulation of circ-ZEB1 inhibits proliferation and tumor growth of non-small cell lung cancer (NSCLC). (A) RT-qPCR shows the expression of circ-ZEB1 in A549 and PC9 cells following transfection with negative control (NC) and circ-ZEB1 silencing (sh-circ-ZEB1) vectors. Data are expressed as mean ± standard deviation (SD). *⁣*^*∗∗∗*^*p* < 0.001 versus NC. (B and C) Cell counting kit-8 (CCK8) assays show the proliferation of A549 and PC9 cells. Data are expressed as mean ± SD. *⁣*^*∗∗∗*^*p* < 0.001 versus NC. (D and E) 5-Ethynyl-20-deoxyuridine (EdU) assay shows the proliferation of A549 and PC9 cells. Data are expressed as mean ± SD. *⁣*^*∗∗∗*^*p* < 0.001 versus NC. (F) Representative photographs of A549 tumor formation in xenografts of nude mice. (G) Summary of tumor volumes. Data are expressed as mean ± SD. *⁣*^*∗∗∗*^*p* < 0.001 versus NC. (H) Summary of tumor weight in mice. Data are expressed as mean ± SD. *⁣*^*∗∗∗*^*p* < 0.001 versus NC. (I and J) Immunohistochemical detection shows the percentage of Ki67-positive cells. The relative number of Ki67-positive cells was calculated. Data are expressed as mean ± SD. *⁣*^*∗∗∗*^*p* < 0.001 versus NC.

RT-qPCR showed that circ-ZEB1 expression was decreased in A549 and PC9 cells posttransfection with siRNA targeting circ-ZEB1 (sh-circ-ZEB1; Figure 2A). CCK8 (Figure 2B,C) and EdU (Figure 2D,E) assays showed that circ-ZEB1 silencing suppressed proliferation of A549 and PC9 cells. Tumor volume and weight were reduced in nude mice injected with A549 cells following sh-circ-ZEB1 transfection (Figure 2F–H). Immunohistochemical detection showed that circ-ZEB1 silencing suppressed Ki67 expression in tumor tissues (Figure 2I,J), suggesting circ-ZEB1 downregulation inhibits NSCLC cell proliferation.

### 3.3. Downregulation of circ-ZEB1 Inhibits NSCLC Cell Migration and Tumor Lung Metastasis

**Figure 3:**
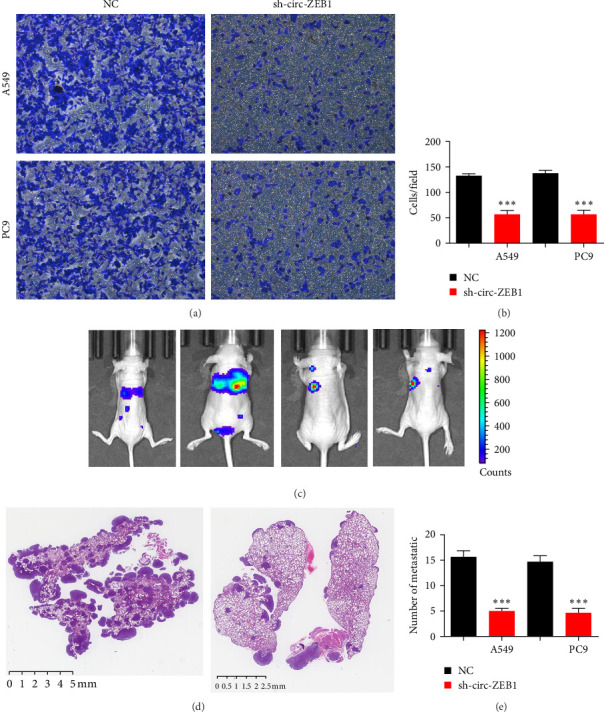
Downregulation of circ-ZEB1 inhibited non-small cell lung cancer (NSCLC) cell migration and tumor lung metastasis. (A and B) Transwell assay shows the migration of A549 and PC9 cells. Data are expressed as mean ± standard deviation (SD). *⁣*^*∗∗∗*^*p* < 0.001 versus NC. (C) Live imaging shows the pulmonary metastasis of Luc-A549 cells. (D and E) Numbers of metastatic foci in lung tissues were determined based on hematoxylin and eosin (HE) staining. Data are expressed as mean ± SD. *⁣*^*∗∗∗*^*p* < 0.001 versus NC.

Transwell assay showed that circ-ZEB1 downregulation suppressed migration of PC9 and A549 cells (Figure 3A,B). Living image detection demonstrated pulmonary metastasis of A549 cells, revealing that circ-ZEB1 silencing decreased pulmonary metastasis by decreasing metastatic foci counts among lung tissues (Figure 3C–E). These results indicate downregulation of circ-ZEB1 inhibits NSCLC invasion.

### 3.4. miR-491-5p and EIF5A are Downstream Targets of circ-ZEB1

**Figure 4:**
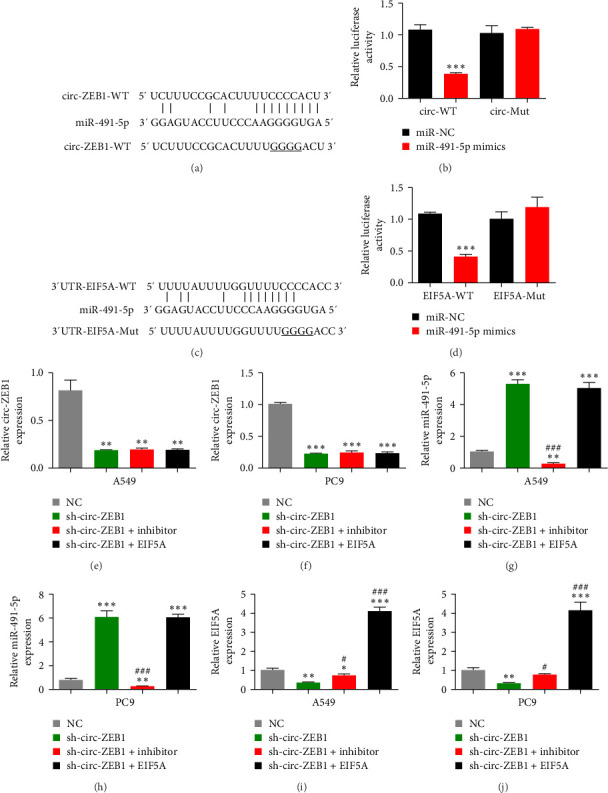
miR-491-5p and EIF5A are downstream targets of circ-ZEB1. (A and B) Luciferase activity of circ-ZEB1 in A549 cells transfected with miR-491-5p mimics, which were putative binding sites for the circ-ZEB1 sequence. Luciferase activity was normalized with Renilla luciferase. (C and D) Bioinformatics analysis shows putative downstream targets of miR-491-5p determined using different websites. Relative luciferase activity determined 48 h following transfection of A549 cells with miR-491-5p mimic/NC or EIF5A-3′ untranslated region (UTR) wild type/mutant (WT/Mut). Data are expressed as mean ± standard deviation (SD). *⁣*^*∗∗∗*^*p* < 0.001. (E–J) RT-qPCR shows the expression of circ-ZEB1, miR-491-5p, and EIF5A in A549 and PC9 cells transfected with sh-circ-ZEB1, miR-491-5p inhibitor, and EIF5A-overexpressing single or multiple vectors. Data are expressed as mean ± SD. *⁣*^*∗*^*p* < 0.05, *⁣*^*∗∗*^*p* < 0.01, and *⁣*^*∗∗∗*^*p* < 0.001 versus NC. ^#^*p* < 0.05 and ^###^*p* < 0.001 versus sh-circ-ZEB1.

Bioinformatics analysis showed that miR-491-5p is a downstream target of circ-ZEB1 (Figure 4A). Transfection of a luciferase reporter vector with miR-491-5p mimic validated that miR-491-5p significantly reduced fluorescein intensity, indicating miR-491-5p is a downstream target of circ-ZEB1 (Figure 4B).

Bioinformatics outputs revealed that EIF5A is a downstream target of miR-491-5p. The Mut or Wt 3′ UTR-EIF5A sequences, including the miR-491-5p-binding sequence, were cloned into the luciferase reporter vector to further validate associations between EIF5A and miR-491-5p (Figure 4C). The luciferase reporter vectors were transfected into A549 cells with or without the miR-491-5p mimic. miR-491-5p suppressed luciferase activity in WT but not Mut cells (Figure 4D).

RT-qPCR showed that circ-ZEB1 expression was decreased following circ-ZEB1 silencing. Downregulation of miR-491-5p or overexpression of EIF5A had no effects on circ-ZEB1 expression in both A549 and PC9 cells (Figure 4E,F), suggesting miR-491-5p and EIF5A are downstream of circ-ZEB1. RT-qPCR also showed that circ-ZEB1 silencing increased miR-491-5p expression. EIF5A overexpression had no effects on sh-circ-ZEB1-induced miR-491-5p expression (Figure 4G,H), suggesting miR-491-5p is downstream of circ-ZEB1. Our data showed that circ-ZEB1 silencing reduced EIF5A expression. Moreover, suppressing miR-491-5p reversed the effects of sh-circ-ZEB1 on EIF5A expression. Transfection of an EIF5A-overexpressing vector significantly increased EIF5A expression (Figure 4I,J), indicating circ-ZEB1 promotes EIF5A expression through miR-491-5p sponging.

### 3.5. EIF5A Overexpression and miR-491-5p Suppression Reversed NSCLC Cell Migration and Proliferation Post circ-ZEB1 Silencing

**Figure 5:**
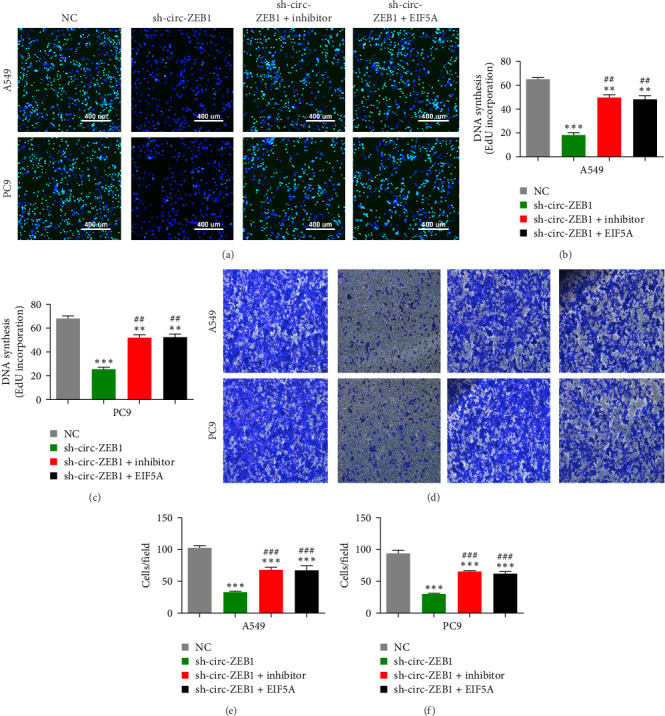
Overexpression of EIF5A or inhibition of miR-491-5p reversed non-small cell lung cancer (NSCLC) cell proliferation and migration after circ-ZEB1 silencing. (A–C) 5-Ethynyl-20-deoxyuridine (EdU) assay shows the proliferation of A549 and PC9 cells. Data are expressed as mean ± standard deviation (SD). *⁣*^*∗∗*^*p* < 0.01 and *⁣*^*∗∗∗*^*p* < 0.001 versus NC. ^##^*p* < 0.001 versus sh-circ-ZEB1. (D–F) Transwell assay shows the invasion and migration of A549 and PC9 cells. Data are expressed as mean ± SD. *⁣*^*∗∗∗*^*p* < 0.001 versus NC. ^###^*p* < 0.001 versus sh-circ-ZEB1.

EdU detection showed that EIF5A overexpression and miR-491-5p suppression restored NSCLC cell proliferation post circ-ZEB1 silencing (Figure 5A–C). Transwell migration assay showed that EIF5A overexpression and miR-491-5p suppression restored migration of both A549 and PC9 cells post circ-ZEB1 silencing (Figure 5D–F).

## 4. Discussion

Previous studies have shown that circ-ZEB1 expression is increased in liver cancer. circ-ZEB1 could enhance PIK3CA expression via miR-199a-3p silencing and affecting liver cancer progression [12]. circ-ZEB1 expression has been shown to be increased in NSCLC tissues and cells. hsa_circ_0018082 (circ-ZEB1) was produced by cyclizing exons from the ZEB1 gene located on chr10:31607423-31676195. ZEB1 is 68,772 bp; the spliced mature circRNA is also 68,772 bp. Thus, hsa_circ_0018082 is also called circ-ZEB1. The study also found that silencing circ-ZEB1 inhibited NSCLC cell migration and proliferation indicating circ-ZEB1 plays an important role in NSCLC progression.

Several studies have shown that circRNAs regulate gene expression by miRNA sponging [13–15]. The present study employed bioinformatics analysis to examine whether circ-ZEB1 acted with miRNAs; luciferase reporter analysis revealed miR-491-5p as a downstream target of circ-ZEB1. Previous studies have shown that high miR-491-5p expression reduced migration, invasion, and proliferation in different cancers, including pancreatic cancer, breast cancer, and colorectal cancer [16–20]. miR-491-5p expression is decreased in NSCLC [21] and has been shown to suppress NSCLC cell migration and proliferation [22, 23]. This study found that circ-ZEB1 silencing enhanced miR-491-5p expression. Inhibiting miR-491-5p restored cell migration and proliferation post circ-ZEB1 silencing.

Other research has revealed that miR-491-5p may interact with the 3′ UTR of EIF5A. EIF5A expression was decreased in NSCLC tumors [23, 24]. Increased EIF5A expression was positively correlated with higher *T*, *N*, pathologic stage, and poor primary therapeutic outcome [25, 26]. circ-ZEB1 silencing downregulated EIF5A expression. High expression of EIF5A promotes the epithelial–mesenchymal transition [27]. EIF5A can regulate PEAK1-YAP1-TEAD signaling and cancer progression [28]. Overexpression of EIF5A increased cell proliferation and migration post circ-ZEB1 silencing. Further investigations revealed that miR-491-5p overexpression suppressed EIF5A expression. EIF5A overexpression also increased cell proliferation and migration post miR-491-5p upregulation. We also found that EIF5A overexpression and miR-491-5p inhibition reversed malignant NSCLC progression following silencing of circ-ZEB1.

Overall, this study revealed that circ-ZEB1 increased EIF5A expression by sponging miR-491-5p to exert tumorigenic effects. But the regulatory mechanism of EIF5A to the progress of NSCLC still largely unclear and need for the further study. Thus, we have identified a circ-ZEB1/miR-491-5p/EIF5A axis and biological effects in malignant NSCLC. This axis may be useful as a candidate therapeutic marker of NSCLC.

## Data Availability

The datasets used and/or analyzed during the current study are available from the corresponding author on reasonable request.
